# The association between short-term response and long-term survival for cervical cancer patients undergoing neoadjuvant chemotherapy: a system review and meta-analysis

**DOI:** 10.1038/s41598-018-19948-0

**Published:** 2018-01-24

**Authors:** Shi-yi Kong, Kecheng Huang, Chao Zeng, Xiangyi Ma, Shixuan Wang

**Affiliations:** Department of Obstetrics and Gynecology, Tongji Hospital, Tongji Medical College, Huazhong University of Science and Technology, Wuhan, Hubei China

## Abstract

Controversy exists regarding whether a short-term response has an impact on the long-term survival of cervical cancer patients undergoing neoadjuvant chemotherapy (NACT). This study was designed to identify the predictive role of an early response by pooling the results of previous studies. The PubMed and Embase databases were searched through July 2016, and the associations between an early response and disease-free survival (DFS) were pooled by hazard ratio (HR) using random effects models. Six studies involving 490 cervical cancer patients, with 336 responders and 154 non-responders, were finally included in the meta-analysis. The HR for 1-year DFS between early responders and non-responders was 0.25 (95% CI 0.10–0.58, P = 0.001). The HRs for 2-, 3-, 4-, and 5-year DFS were 0.28 (95% CI 0.15–0.56), 0.27 (95% CI 0.16–0.45), 0.29 (95% CI 0.17–0.50) and 0.33 (95% CI 0.20–0.54), respectively. No obvious heterogeneity was found among the studies, with I^2^ = 0, and a sensitivity analysis showed that all pooled results were robust with logHR confidence limits < 0. An early response was associated with DFS, and responders achieved a significantly higher survival rate than non-responders. This finding should be validated in future prospective studies.

## Introduction

Cervical cancer is a common malignant tumor disease in females worldwide. According to the latest results, more than 527,600 new cases and 265,700 deaths were estimated to be attributed to this disease in 2012^1^. Although concurrent chemo-radiotherapy (CCRT) is the traditional treatment, the side effects in women are severe, especially for young patients^2,3^. Additionally, patients have become increasingly younger in recent decades. Considering the severe side effects of CCRT, great efforts have been made to develop new therapeutic drugs and devices. In recent decades, doctors and clinicians have resorted to neoadjuvant chemotherapy (NACT) as an alternative treatment for cervical cancer^4^.

Previous studies have shown that NACT can help to reduce tumor size and cancer cell metastasis, thus making the malignant disease operable^5^. Many patients choose NACT plus surgery rather than radiotherapy to avoid sacrificing their quality of life; these patients can maintain vaginal and ovarian function, as well as their pelvic organ function. These advantages are particularly important for young or pregnant women who wish to preserve their fertility^6^. For patients with early stage cervical cancer, NACT can result in less invasive surgery. Using NACT, minimally invasive surgery, such as cervical conization and radical trachelectomy, can be performed to spare the uterus and reproductive function^7^.

Although a short-term response after treatment may allow patients to choose a more effective treatment regimen in many malignant tumor diseases^8^, the predictive role of the short-term response to NACT on long-term survival is still unclear for cervical cancer patients^9,10^. This study was designed to identify the prognostic role of a short-term response on the overall survival of cervical cancer patients submitted to NACT.

## Results

### Literature search

**Figure 1:**
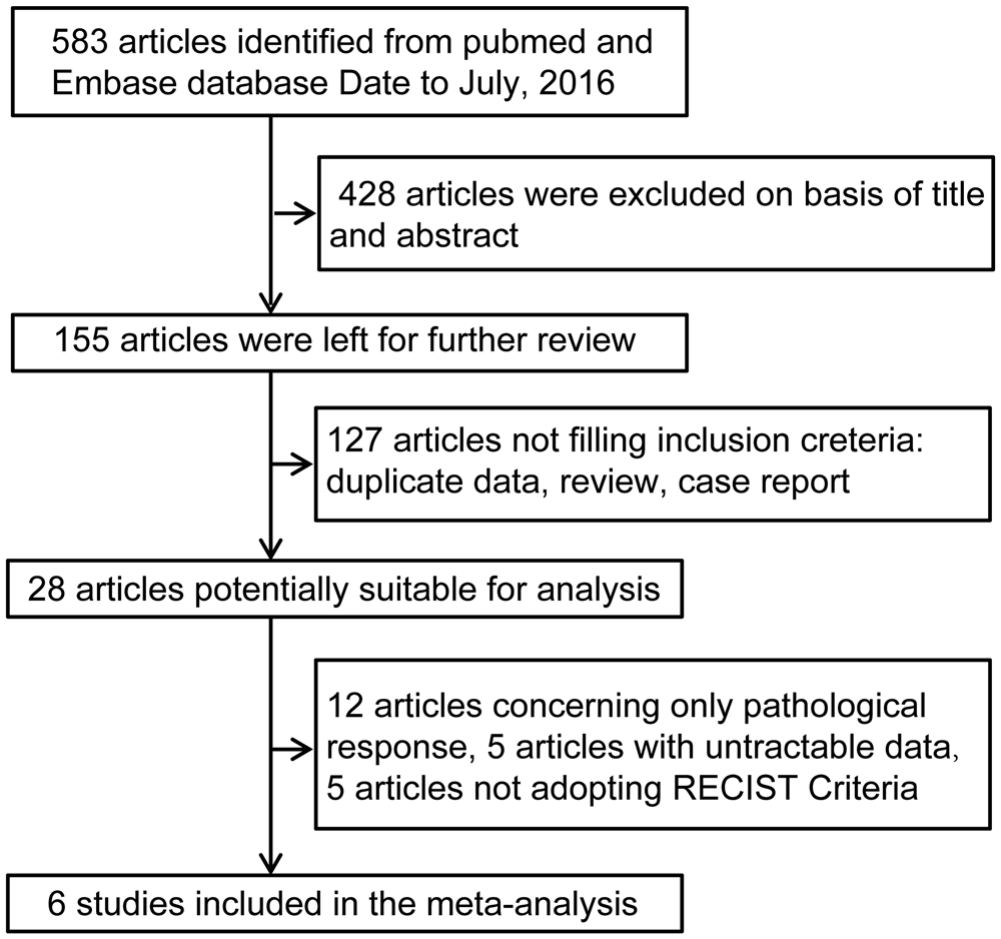
Flow chart of the meta-analysis. Studies using RECIST criteria were included; otherwise, they would be excluded. RECIST indicates Response Evaluation Criteria in Solid Tumor.

A total of 583 articles were found via searches using the key words described in the methods section (Fig. 1). During the first round of screening, 428 articles were excluded after reviewing the titles and abstracts. During the second round of screening, 127 articles were excluded, as these articles were all case reports or reviews. During the third round of screening, 22 articles were excluded for reasons such as duplicated data, intractable data with neither the HR nor the survival curve reported; papers were also excluded in which the RECIST criteria were not adopted. After all three rounds of screening, 6 articles were included for further evaluation and were eventually included in the combined analysis.

### Relationship between the clinical response and disease-free survival (DFS)

#### Characteristics of the studies

The details of the included studies are listed in Table 1. The table reveals the association between the clinical response and DFS, either with adjustment of parameters or alone. The 6 included studies consisted of 490 patients, which included 336 clinical responders and 154 clinical non-responders. All 6 studies were conducted in East Asian areas.

**Table 1:** Characteristics of studies included in the meta-analysis.

Study	Country	Study period	No. of cases (non-responders)	No. of all patients	Adjustment	Follow-up period
Xie^17^	China	2003–2008	18	52	Tumor size, the expression of ALDH1	3–123 months
Park^19^	Korea	1997–2007	15	43	Node, the expression of ERCC1	6–139 months
Liu^14^	China	2002–2011	40	103	None	6–113 months
Yang^18^	China	2007–2012	33	115	None	6–75 months
Li^12^	China	2000–2011	43	154	None	6–142 months
Shoji^10^	Japan	2002–2011	5	23	None	9–90 months

#### 1-year HR

**Figure 2:**
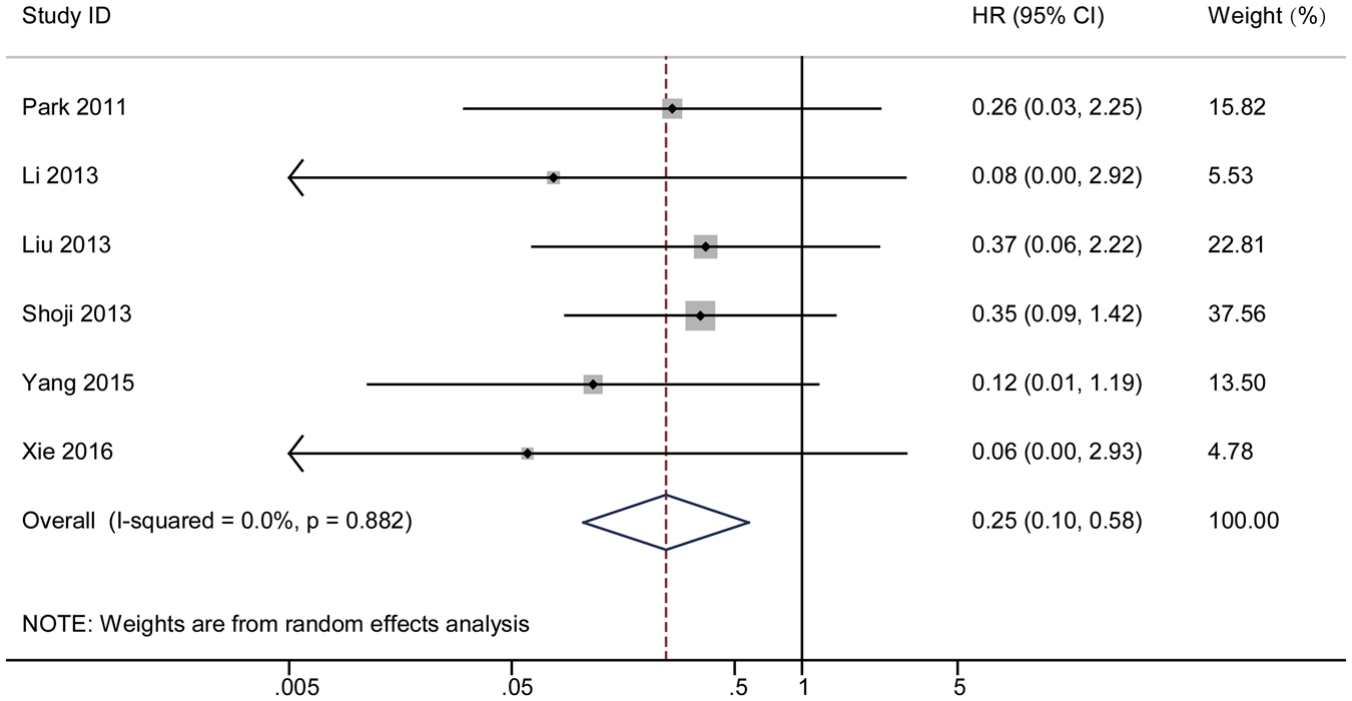
The pooled 1-year HR (hazard ratio) of non-responder with overall survival among cervical cancer patients undergoing neoadjuvant chemotherapy. The summary estimates were obtained by using a random-effects model. The data markers indicate the HRs comparing non-responder with responder. The size of the data markers indicates the weight of the study, which is the inverse variance of the effect estimate. The diamond data markers indicate the pooled HRs. CI indicates confidence interval.

**Figure 3:**
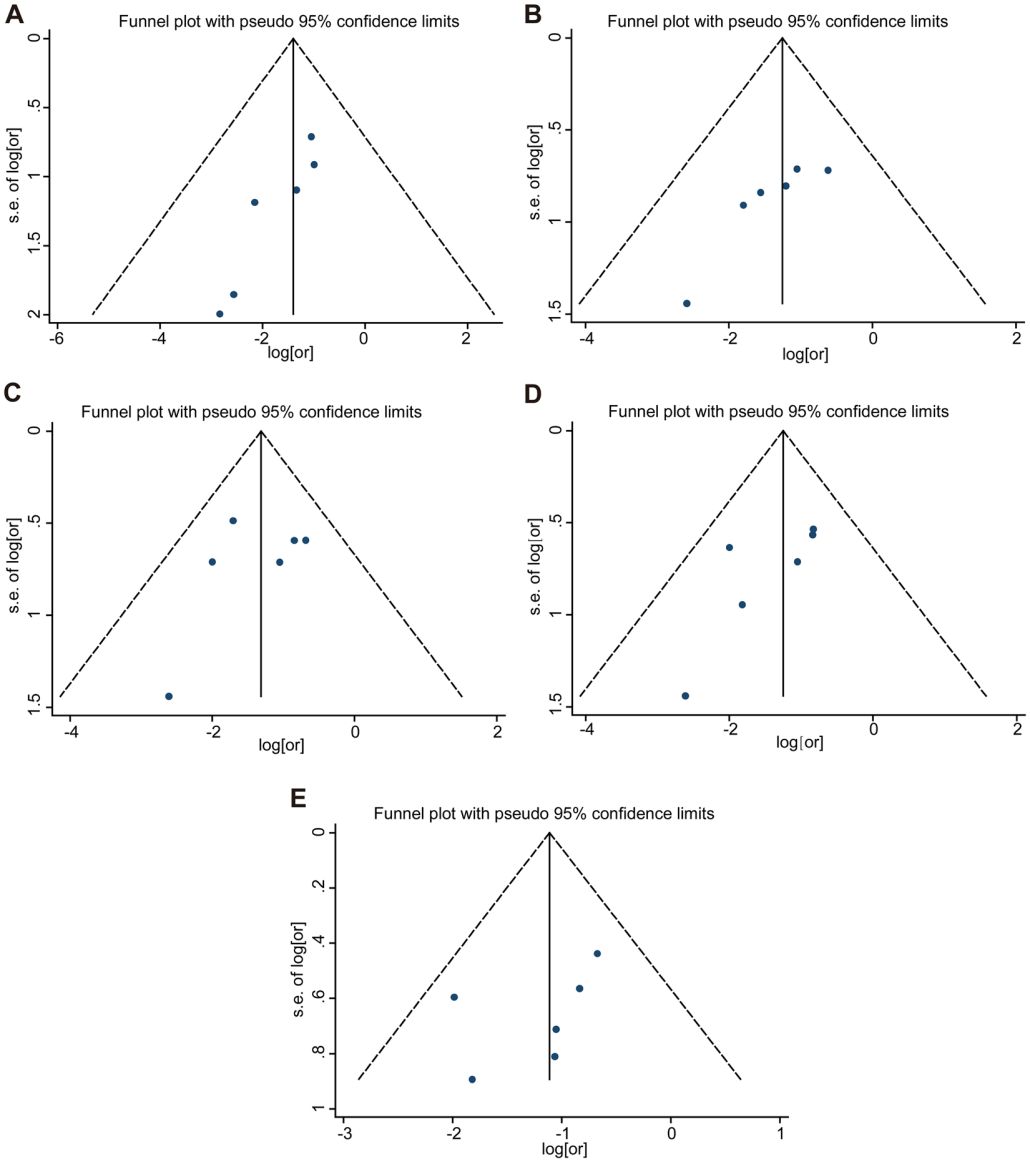
Funnel plots for detection of publication bias. The pseudo 95% confidence interval (CI) is computed as part of the analysis that produces the funnel plot, and corresponding to the expected 95% CI for a given standard error (SE). HR indicates hazard ratio. From A to E, it represents 1-year survival to 5-year survival respectively.

A forest plot was employed to illustrate the association between a short-term response and overall survival. The HR of each study was determined and is listed in Fig. 2. The dots in the middle of the bar indicate the HR, and the spread of the bars indicates the 95% CI of the HR. The diamond in each bar indicates the corresponding weight of the included study. The pooled result after the combination of the studies is shown at the bottom of the forest plot. The analysis showed a combined result with an HR = 0.25 and a 95% CI of 0.10–0.58. A Cochrane *Q* test produced a *P* value of 0.882 and an I^2^ equal to 0%. A funnel plot was constructed to visually demonstrate the probability of publication bias (Fig. 3A). Non-parametric and parametric tests were also employed to detect publication bias (Supplementary Figure S1). A sensitivity analysis was used to determine whether heterogeneity existed in the combined analysis, which is shown in Supplementary Figure S2.

#### 2-year HR

**Figure 4:**
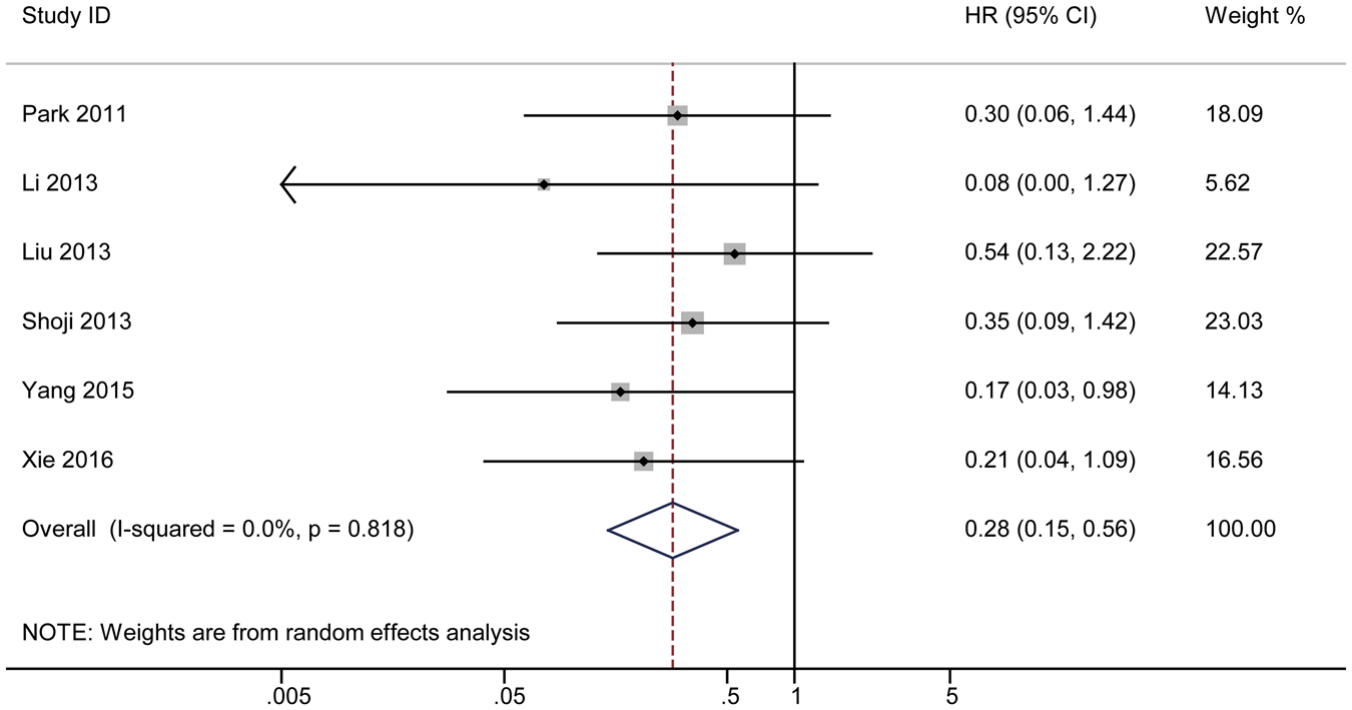
The pooled 2-year HR of non-responder with overall survival among cervical cancer patients undergoing neoadjuvant chemotherapy. The summary estimates were obtained by using a random-effects model. The data markers indicate the HRs comparing non-responder with responder. The size of the data markers indicates the weight of the study, which is the inverse variance of the effect estimate. The diamond data markers indicate the pooled HRs. CI indicates confidence interval.

The combined results for the second year are also shown by forest plot in Fig. 4. The plot shows an HR of 0.28 with a 95% CI of 0.15–0.56. A Cochrane *Q* test was also performed to test the possible heterogeneity in the analysis (*P* = 0.818) with I^2^ = 0. A funnel plot was constructed to visually reveal the bias (Fig. 3B). Begg’s and Egger’s tests were also used to calculate the actual *P* value with non-parametric and parametric methods (Supplementary Figure S3). A sensitivity analysis was used to detect the heterogeneity in the combined analysis (Supplementary Figure S4). Each study was excluded individually, and the results of the remaining studies were pooled. Each combined HR was calculated individually.

#### 3-year HR

**Figure 5:**
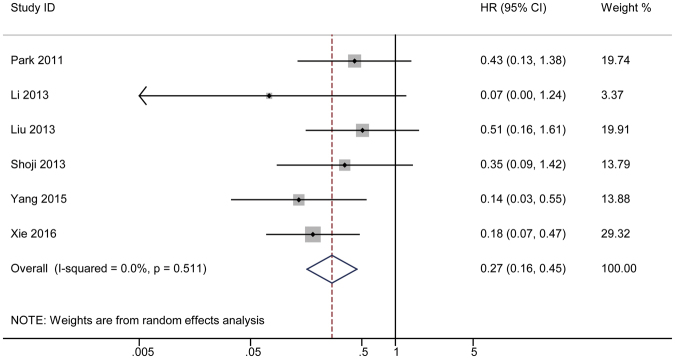
The pooled 3-year HR of non-responder with overall survival among cervical cancer patients undergoing neoadjuvant chemotherapy. The summary estimates were obtained by using a random-effects model. The data markers indicate the HRs comparing non-responder with responder. The size of the data markers indicates the weight of the study, which is the inverse variance of the effect estimate. The diamond data markers indicate the pooled HRs. CI indicates confidence interval.

**A** forest plot was created to show the combined DFS in the third year (Fig. 5). The analysis showed a final pooled HR = 0.27 for the 3-year DFS with a 95% CI of 0.16–0.45. A Cochrane *Q* test showed that *P* = 0.511; I^2^ test revealed a value of 0. A funnel plot (Fig. 3C) was constructed, and Begg’s and Egger’s tests (Supplementary Figure S5) were performed to observe the publication bias. A sensitivity analysis was also performed to test whether the result of the combined analysis was robust (Supplementary Figure S6).

#### 4-year HR

**Figure 6:**
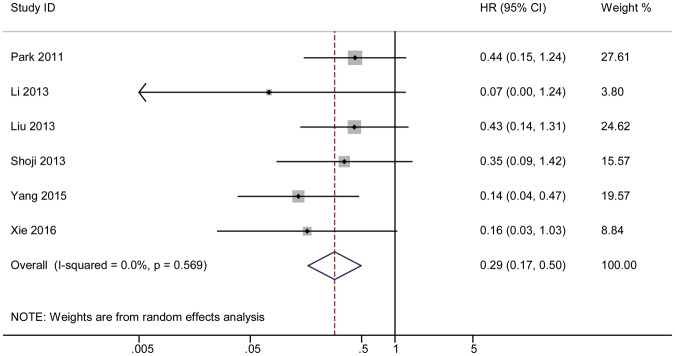
The pooled 4-year HR of non-responder with overall survival among cervical cancer patients undergoing neoadjuvant chemotherapy. The summary estimates were obtained by using a random-effects model. The data markers indicate the HRs comparing non-responder with responder. The size of the data markers indicates the weight of the study, which is the inverse variance of the effect estimate. The diamond data markers indicate the pooled HRs. CI indicates confidence interval.

Using the same methods described above, a forest plot showed a final combined HR in the fourth year of 0.29, and the corresponding 95% CI was 0.17–0.50. A Cochrane *Q* test produced a P value of 0.569 and I^2^ test showed a value of 0 (Fig. 6). A funnel plot was also constructed (Fig. 3D). The results of the Begg’s and Egger’s tests are shown in Supplementary Figure S7. A sensitivity analysis was performed to show the distribution of the combined results by excluding each study individually (Supplementary Figure S8).

#### 5-year HR

**Figure 7:**
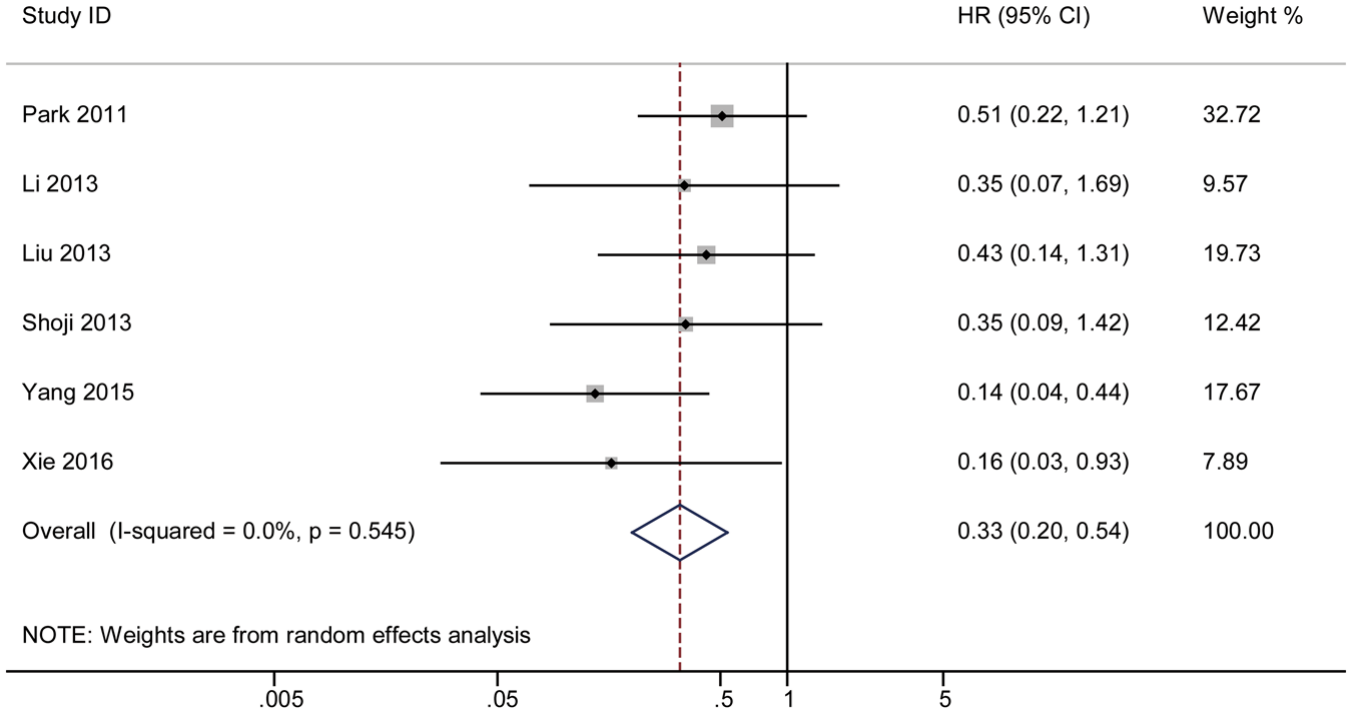
The pooled 5-year HR of non-responder with overall survival among cervical cancer patients undergoing neoadjuvant chemotherapy. The summary estimates were obtained by using a random-effects model. The data markers indicate the HRs comparing non-responder with responder. The size of the data markers indicates the weight of the study, which is the inverse variance of the effect estimate. The diamond data markers indicate the pooled HRs. CI indicates confidence interval.

The combined HR for the fifth year was also determined and shown by a forest plot (HR = 0.33 and 95% CI 0.20–0.54). A Cochrane *Q* test revealed *P* = 0.545 (Fig. 7) while I^2^ test showed a value of 0. A funnel plot was constructed (Fig. 3E), and Begg’s and Egger’s tests were conducted to investigate the publication bias (Supplementary Figure S9). A Sensitivity analysis was also conducted to test the robustness of the combined results (Supplementary Figure S10).

## Discussion

By combining previous study results, the present study found that a short-term response was significantly associated with the long-term survival of cervical cancer patients who underwent NACT. Additionally, overall survival may be partly predicted by the short-term response when it is evaluated by the RECIST criteria.

Our findings validated several previous studies in which the predictive role of the short-term response was also evaluated among cervical cancer patients. Chen and colleagues performed a randomized controlled trial (RCT) on 142 cervical cancer patients who underwent NACT from 1999 to 2003. They found that the response to NACT was an independent prognostic factor of long-term survival after adjustment for age, International Federation of Gynecology and Obstetrics (FIGO) stage, pathological grade, histological type, tumor size, lymph node metastasis, and parametrial infiltration. Cai and colleagues also performed a prospective RCT of 106 patients from 1999 to 2005, and they found that responders achieved a better survival rate than non-responders^11^. Li and colleagues performed a study on 304 patients in 2012, and they similarly found that responders achieved higher survival rates than non-responders^12^. Other studies that used the WHO criteria have also shown similar results indicating that a clinical response was associated with better long-term survival. In 2011, Xiong and colleagues conducted a retrospective study and demonstrated that the response to NACT was associated with long-term survival^13^. Our results were slightly different from those of Liu and colleagues, who found that a short-term response did not lead to a significantly higher survival rate^14^. We speculate that the statistical power of that study may not be sufficient to provide a definite conclusion, considering the number of subjects enrolled in the study^15^. Thus, we hypothesize that if the study population was larger, a significant difference would have been observed.

The predictive effect of the short-term response on long-term survival has always been a focus of research of solid tumors, as it may highlight a method for personalized treatment. The short-term response can be observed a very short time after chemotherapy, and the role of chemotherapy drugs may be quickly determined by doctors and patients. Accordingly, patients can be administered the most effective treatment regimens. A proper treatment regimen may help patients to achieve longer survival, and it may also help to decrease the cost of medical treatment.

Our study has some limitations. First, we did not pool individual data, which could have provided a more accurate result. Second, the difference in the survival rate between responders and non-responders was not investigated using WHO criteria in this study. Therefore, in future studies, we plan to collect individual data to obtain a more accurate result. We also plan to determine new methods to calculate the HR according to the WHO criteria.

In conclusion, we performed a combined analysis of the predictive role of the short-term response on long-term survival for cervical cancer patients who underwent NACT. We found that clinical responders achieved higher survival rates than non-responders. This finding may help doctors to evaluate the survival of this group of patients and may help to determine more effective treatment methods. Future RCTs should be performed to validate our results and to provide clear conclusions with less bias.

## Methods

### Literature search

In August 2016, a search for literature in this field was performed in the PubMed and Embase databases by two doctors independently. The key words used for the search included the following: “NACT” or “neoadjuvant chemotherapy” or “preoperative chemotherapy”, plus “cervical carcinoma” or “cervical cancer” or “uterine cervical neoplasms”, plus “responding” or “response” or “clinical response” or “responder” or “remission” or “responsiveness”. To include as many eligible articles as possible, we also reviewed the reference lists of the retrieved articles.

### Study identification

Inclusion criteria. To determine their eligibility, two reviewers independently reviewed the titles and abstracts of the articles (S.Y.K. and K.C.H.). The selected articles were required to be original research articles. The articles were written in English and published in a peer-reviewed journal in a relevant discipline. All cases in the articles were cervical carcinoma patients with a definite diagnosis. Using the Newcastle-Ottawa Scale (NOS), our team performed a quality assessment of the included studies, as described in a previous study^16^.

Exclusion criteria. In the primary search, a total of 583 papers were retrieved. After reading the titles and abstracts, we excluded 428 articles from further analysis due to irrelevance to the present research. Then, we excluded articles that did not adopt the RECIST criteria; articles that were only concerned with the pathological response and not the clinical response were also excluded from further analysis; studies with only descriptive results but without statistical data were also excluded. These studies were carefully reviewed to exclude duplicated information. Finally, 6 articles were included in the present study, and these 6 studies were used for the final analysis^10,12,14,17–19^.

### Statistical analyses

According to the RECIST criteria, clinical responders included individuals with a complete response (CR) or partial response (PR), while clinical non-responders included those with stable disease (SD) or progressive disease (PD). The RECIST criteria are a widely used standard for evaluating the short-term response of solid tumors^20^.

The hazard ratio (HR) and 95% CI were the most common statistics used across the studies to measure the association between the short-term response and survival^10^. When this information could not be obtained from the articles, Engauge Digitizer software was used to determine the survival curve of the included studies^21^ based on the calculus theory and integral theory^22,23^. The pooling process of the HR and its corresponding 95% CI was visually illustrated by forest plots. During pooling, a Cochrane *Q* test was employed to test the heterogeneity; the significance level was set at *P* < 0.10, according to a previous study^24^. The I^2^ statistic was also used to test the heterogeneity across the studies, and a value of I^2^ > 50% was considered to indicate significant heterogeneity^25^. A random effects model was used to calculate the combined HR according to the DerSimonian and Laird method^26^. The possibility of publication bias was evaluated by visual screening of a funnel plot, and both Begg’s test and Egger’s test were used to evaluate the publication bias^27,28^. A sensitivity analysis was conducted to evaluate the robustness of the combined results^24^. During our research, one study was omitted at a time to test the robustness of the combined results. Stata version 11 (Stata Corp, College Station, TX) was used for the statistical analysis. Differences with a two-sided value of *P* < 0.05 were considered statistically significant.

## Electronic supplementary material

Supplementary material
