# Supplementary material for: The association between short-term response and long-term survival for cervical cancer patients undergoing neoadjuvant chemotherapy: a system review and meta-analysis
Source: Sci Rep. 2018 Jan 24;8:1545. doi: 10.1038/s41598-018-19948-0 (PMC5784110; doi:10.1038/s41598-018-19948-0)
Supplement: Supplementary file 1 — Supplementary material [file 41598_2018_19948_MOESM1_ESM.pdf]

The association between short-term response and long-term survival for cervical cancer patients undergoing neoadjuvant chemotherapy: a system review and meta-analysis

Shi-yi Kong[*], Kecheng Huang[*], Chao Zeng, Xiangyi Ma[#] & Shixuan Wang[#]

Supplementary Fig. S1. Title: Begg's and Egger's publication bias plot for HR of 1-year OS.

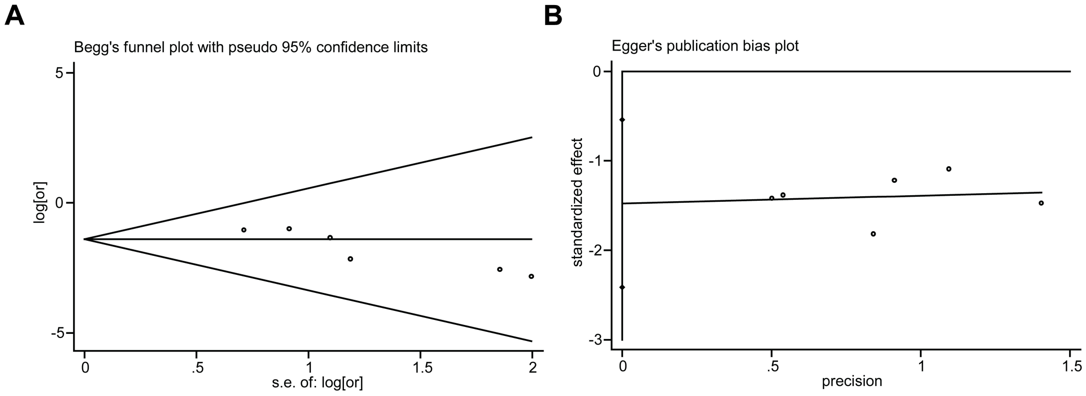

Legend: (A) The pseudo 95% confidence interval (CI) is computed as part of the analysis that produces the funnel plot, and corresponding to the expected 95% CI for a given standard error (SE) (P = 0.02). (B) Publication bias can be concluded by intercept as well as p-value (P = 0.01). HR indicates hazard ratio.

Supplementary Fig. S2. Title: Sensitivity analysis for testing the robust of HR of 1-year OS.

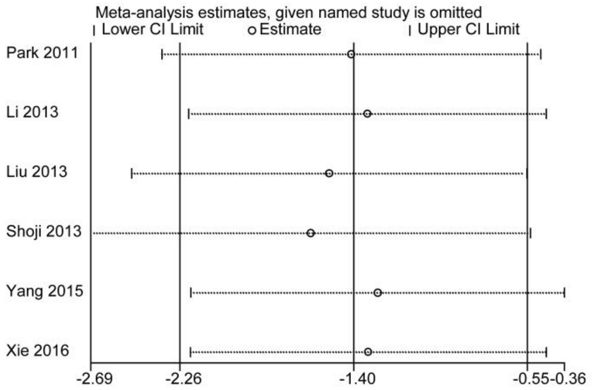

Legend: The small circle indicates the estimated logHR, given the named study is omitted. Accordingly, the bar is corresponding to the lower limit of 95% CI of the logHR. The Figure indicates that the result is robust.

Supplementary Fig. S3. Title: Begg's and Egger's publication bias plot for HR of 2-year OS.

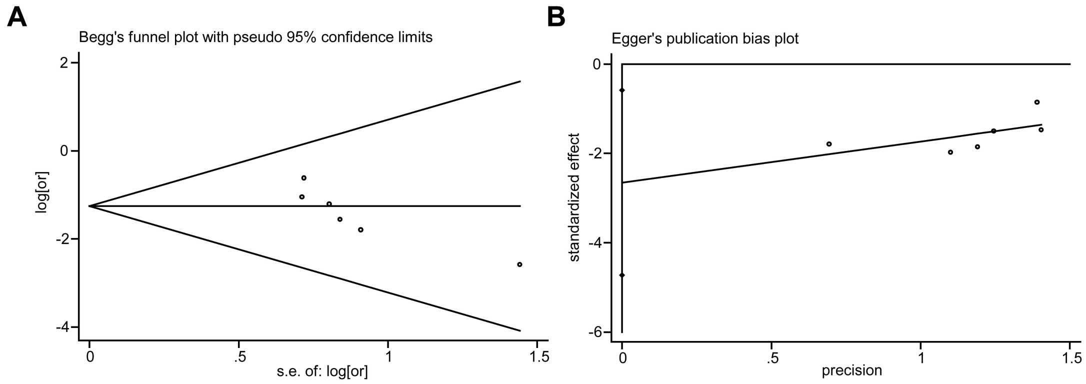

Legend: (A) The pseudo 95% confidence interval (CI) is computed as part of the analysis that produces the funnel plot, and corresponding to the expected 95% CI for a given standard error (SE) (P = 0.02). (B) Publication bias can be concluded by intercept as well as p-value (P = 0.02). HR indicates hazard ratio.

Supplementary Fig. S4. Title: Sensitivity analysis for testing the robust of HR of 2-year OS.

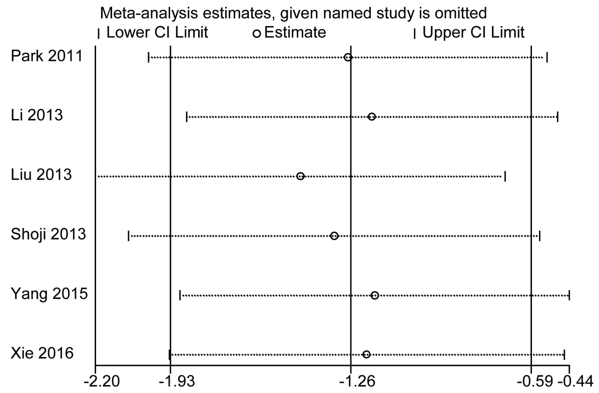

Legend: The small circle indicates the estimated logHR, given the named study is omitted. Accordingly, the bar is corresponding to the lower limit of 95% CI of the logHR. The Figure indicates that the result is robust.

Supplementary Fig. S5. Title: Begg's and Egger's publication bias plot for HR of 3-year OS.

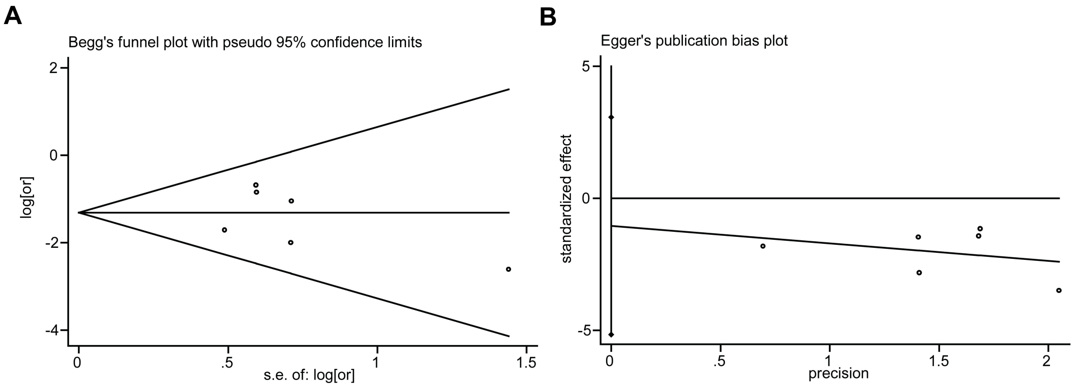

Legend: (A) The pseudo 95% confidence interval (CI) is computed as part of the analysis that produces the funnel plot, and corresponding to the expected 95% CI for a given standard error (SE) (P = 0.71). (B) Publication bias can be concluded by intercept as well as p-value. The test showed that no obvious publication bias was observed with P = 0.52. HR indicates hazard ratio.

Supplementary Fig. S6. Title: Sensitivity analysis for testing the robust of HR of 3-year OS.

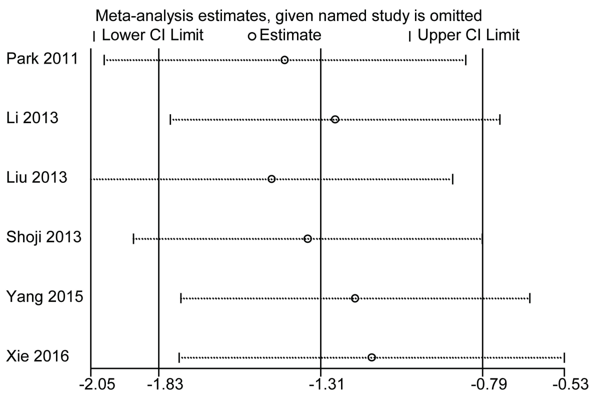

Legend: The small circle indicates the estimated logHR, given the named study is omitted. Accordingly, the bar is corresponding to the lower limit of 95% CI of the logHR. The Figure indicates that the result is robust.

Supplementary Fig. S7. Title: Begg's and Egger's publication bias plot for HR of 4-year OS.

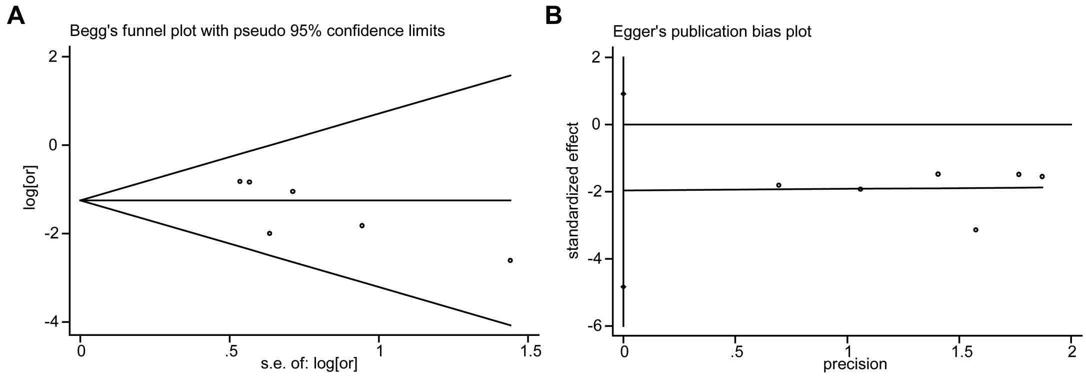

Legend: (A) The pseudo 95% confidence interval (CI) is computed as part of the analysis that produces the funnel plot, and corresponding to the expected 95% CI for a given standard error (SE) (P = 0.13). (B) Publication bias can be concluded by intercept as well as p-value. The test showed that no obvious publication bias was observed with P = 0.13. HR indicates hazard ratio.

Supplementary Fig. S8. Title: Sensitivity analysis for testing the robust of HR of 4-year OS.

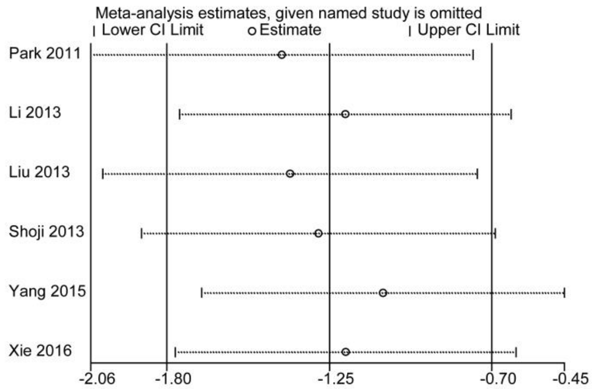

Legend: The small circle indicates the estimated logHR, given the named study is omitted. Accordingly, the bar is corresponding to the lower limit of 95% CI of the logHR. The Figure indicates that the result is robust.

Supplementary Fig. S9. Title: Begg's and Egger's publication bias plot for HR of 5-year OS.

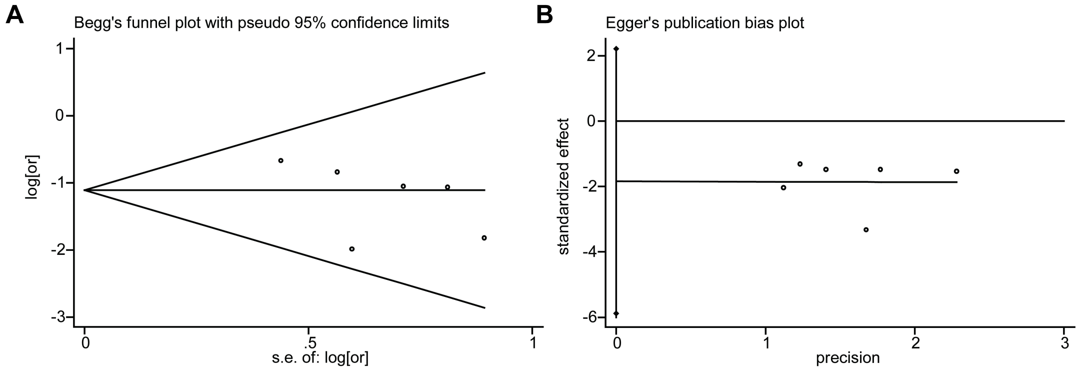

Legend: (A) The pseudo 95% confidence interval (CI) is computed as part of the analysis that produces the funnel plot, and corresponding to the expected 95% CI for a given standard error (SE) (P = 0.13). (B) Publication bias can be concluded by intercept as well as p-value. The test showed that no obvious publication bias was observed with P = 0.28. HR indicates hazard

ratio.

Supplementary Fig. S10. Title: Sensitivity analysis for testing the robust of HR of 5-year OS.

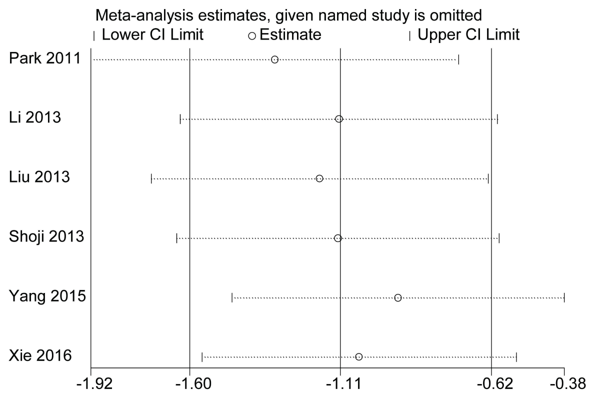

Legend: The small circle indicates the estimated logHR, given the named study is omitted. Accordingly, the bar is corresponding to the lower limit of 95% CI of the logHR. The Figure indicates that the result is robust.
